# Neuropilin Antagonists (NRPas) Block the Phosphorylation of the Cancer Therapeutic Key Factor p38α Kinase Triggering Cell Death

**DOI:** 10.3390/molecules30071494

**Published:** 2025-03-27

**Authors:** Lucia Borriello, Rafika Jarray, Rachel Rignault-Bricard, Matthieu Montes, Nicolas Lopez, Thiago Trovati Maciel, Olivier Hermine, Françoise Raynaud, Luc Demange, Yves Lepelletier

**Affiliations:** 1 Department of Cancer and Cellular Biology, Lewis Katz School of Medicine, Temple University, Philadelphia, PA 19140, USA; lucia-borriello@libero.it; 2 Fox Chase Cancer Center, Cancer Signaling and Microenvironment Program, Philadelphia, PA 19140, USA; 3 Laboratoire de Chimie et Biochimie Pharmacologiques et Toxicologiques (LCBPT), UMR 8601 CNRS, Université Paris Descartes, Sorbonne Paris Cité, UFR Biomédicale des Saints Pères, 45 Rue des Saints Pères, 75270 Paris, CEDEX 06, France; 4 Division of Prions and Related Diseases (SEPIA), CEA (Commissariat à l’Énergie Atomique), Institute of Emerging Diseases and Innovative Therapies (iMETI), 92265 Fontenay-aux-Roses, France; 5 INSERM UMR 1163, Laboratory of Cellular and Molecular Basis of Normal Hematopoiesis and Hematological Disorders: Therapeutical Implications, 24 Boulevard Montparnasse, 75015 Paris, Franceohermine@gmail.com (O.H.); 6 Imagine Institute, Université Paris Cité, 24 Boulevard Montparnasse, 75015 Paris, France; 7 Laboratoire Génomique, Bioinformatique et Chimie Moléculaire, EA7528, Conservatoire National des Arts et Métiers, 292 Rue Saint Martin, 75003 Paris, France; 8 W-MedPhys, 128 Rue la Boétie, 75008 Paris, France; universalphysics@hotmail.com; 9 ENOES/ENESIA, 62 Rue de Miromesnil, 75008 Paris, France; 10 Unité Mixte de Recherche “Institut de Physique Théorique (IPhT)” CEA-CNRS, UMR 3681, Route de l’Orme des Merisiers, 91191 St Aubin-Gif-sur-Yvette, France; 11 UMR 8038 CNRS CiTCoM, Team PNAS, Faculté de Pharmacie, Université Paris Cité, 4 Avenue de l’Observatoire, 75006 Paris, France

**Keywords:** neuropilin-1, neuropilin antagonists, p38α kinase, breast cancer, VEGF

## Abstract

Neuropilin-1 is henceforth a relevant target in cancer treatment; however, its way of action remains partly elusive, and the development of small inhibitory molecules is therefore required for its study. Here, we report that two small-sized neuropilin antagonists (NRPa-47 and NRPa-48), VEGF-A_165_/NRP-1 binding inhibitors, are able to decrease VEGF-Rs phosphorylation and to modulate their downstream cascades in the triple-negative breast cancer cell line (MDA-MB-231). Nevertheless, NRPas exert a divergent pathway regulation of MAPK phosphorylation, such as JNK-1/-2/-3, ERK-1/-2, and p38β/γ/δ-kinases, as well as their respective downstream targets. However, NRPa-47 and NRPa-48 apply a common down-regulation of the p38α-kinase phosphorylation and their downstream targets, emphasising its central regulating role. More importantly, none of the 40 selected kinases, including SAPK2a/p38α, are affected in vitro by NRPas, strengthening their specificity. Taken together, NRPas induced cell death by the down-modulation of pro-apoptotic and anti-apoptotic proteins, cell death receptors and adaptors, heat shock proteins (HSP-27/-60/-70), cell cycle proteins (p21, p27, phospho-RAD17), and transcription factors (p53, HIF-1α). In conclusion, we showed for the first time how NRPas may alter tumour cell signalling and contribute to the down-modulation of the cancer therapeutic key factor p38α-kinase phosphorylation. Thus, the efficient association of NRPas and p38α-kinase inhibitor strengthened this hypothesis.

## 1. Introduction

Neuropilin-1 (NRP-1) and neuropilin-2 (NRP-2) are transmembrane type I glycoproteins sharing 44% sequence homology [1]. Initially, neuropilins (NRPs) were identified as neuronal receptors of specific secreted members of the semaphorin III family involved in axonal guidance and repulsion [2]. Neuropilins are multifunctional non-tyrosine kinase receptors for some VEGF (Vascular Endothelial Growth Factor) family members. VEGF A_165_, a VEGF A spliced form, is upregulated in several tumour tissues, and it was considered to be one of the most efficient pro-angiogenic factors. VEGF A_165_ binds to structurally related tyrosine kinase receptors such as VEGF-R1 (Flt-1), VEGF-R2 (Flk-2) and its NRP co-receptors lacking cytosolic catalytic activity [1,3]. In many cancers, expression of one or both NRPs has been correlated with tumour progression and/or poor prognosis (see for review Prud’homme G.J. and Glinka, 2012, and Wild et al., 2012) [4,5]. Through their direct interactions with VEGF-Rs, NRPs have rapidly emerged as key regulators of angiogenesis and tumour progression and are validated as therapeutic targets [6,7,8].

The drug development against NRP brought the newest tools, such as antibodies [1,9,10], peptides (A7R, EG3287, NRP-1 trans-membrane peptides) [11,12,13,14,15], and peptidomimetics (EG00229, EG01377) [16,17]. Recently, new approaches highlighted the interest of small inhibitory molecules to decrease VEGF binding to NRP and tumour growth in vivo and in vitro [18,19]. In this field, we are the first research team that developed a fully non-peptidic inhibitory molecule, the so-called neuropilin antagonist (NRPa) [20,21]. However, the molecular mechanisms by which NRPs modulate cancer progression are still poorly understood. NRPas should provide additional data for the rational knowledge of the cell signalling involved in tumour development and survival.

In this report, two NRPas, so-called NRPa-47 and NRPa-48, selected from our compound-collection screening [20,22] and from structural docking observations, have been used to unravel the signalling pathways involved in their mechanism of action. Both NRPas induce VEGF-R1 and VEGF-R2 dephosphorylation. These inhibitors rapidly lead to the down-modulation of HIF-1α and VEGF-A_165_ mRNA expression. Surprisingly, NRPa-47 and NRPa-48 have a differential effect on the phosphorylation of MAPKs, AKT, and respective downstream targets. However, both NRPas induce dephosphorylation of the p38α kinase and their downstream targets. Moreover, NRPas induce high oxidative stress reflected by the induction of catalase. Furthermore, this stress should not be regulated by the NRPa-treated cell, which down-regulated the expression of inducible and constitutive heme oxygenases. Taken together, NRPas induced molecular regulation that led to cell death due to the deregulation of pro-apoptotic/anti-apoptotic proteins and, more importantly, by HIF-1α and Survivin down-regulation as well as by the inhibition of the cancer therapeutic key factor p38α.

## 2. Results

### 2.1. Neuropilin Antagonist 47 (NRPa-47) Inhibits VEGF-R1/-R2 Phosphorylation

**Figure 1:**
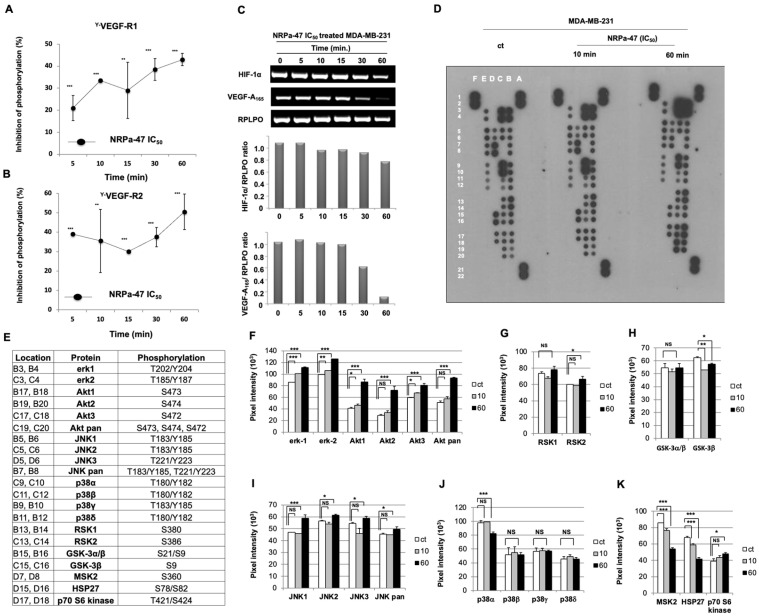
NRPa-47 induces VEGF downstream signalling modification. (**A**,**B**) Total tyrosine VEGF-R1 (Y-VEGF-R1) (**A**) and VEGF-R2 (Y-VEGF-R2) (**B**) phosphorylation inhibition induced by NRPa-47 (IC_50_ = 0.6 µM) on MDA-MB-231 in a time-dependent manner (5 to 60 min) using specific ELISA assays. Curves represent means +/− SD of at least three separate experiments, each performed in triplicate. (**C**) HIF-1α, VEGF-A_165,_ and RPLPO (housekeeping gene) mRNA expressions were assessed in the presence of NRPa-47 in a time course (5 to 60 min). Histograms represent the ratio of HIF-1α/RPLPO and VEGF-A_165_/RPLPO obtained after pixel intensity quantification using ImageJ 1.52q software of this representative experiment. (**D**) ECL developed MAPK protein array film of untreated (control) and NRPa-47-treated (IC_50_ = 0.6 µM) MDA-MB-231 at short time drug exposure (10 and 60 min). The following positions, A1/A2, A21/A22, and F1/F2, were positive controls; E3/E4, E5/E6, E7/E8, E9/10, and E11/E12 were antibody detection controls; and E13/14 were negative controls (background). Images are representative of at least two independent experiments for each condition. (**E**) Location map of phosphorylated proteins targeted in the MAPK protein array and their respective tyrosine (Y), serine (S), and threonine (T) phosphorylation positions detected. (**F**–**K**) Histograms show pixel intensity of untreated (ct, white histograms), NRPa-47-treated MDA-MB-231 at 10 (grey histograms) and 60 min (black histograms) of ERK-1/-2 and AKT-1/-2/-3/-pan (**F**), RSK-1/-2 (**G**), GSK-3α/β and GSK-3β (**H**), JNK-1/-2/-3/-pan (**I**), p38α/p38β/p38γ/p38δ (**J**), MSK2/HSP27/p70S6kinase (**K**) proteins. Histograms represent means ± SD of the previous representative selected experiment analysed using Image-J 1.52q software to quantify pixel intensity. (*, *p* < 0.05; **, *p* < 0.01; ***, *p* < 0.001; NS = not significant).

We previously described a neuropilin antagonist (NRPa), so-called compound-1, that inhibited VEGF-A_165_/NRP-1 binding, tumour survival, and tumour growth in vivo, which is renamed here NRPa-47 [20]. However, the NRPa-47 mechanism of action remained elusive; thus, we focus our present report to describe its function and regulation of cell signalling. As NRP-1 interacts with both VEGF-R1 and VEGF-R2 in the presence of VEGF-A_165_ to mediate intrinsic tyrosine kinase activity, we first studied VEGF-R1/VEGF-R2 phosphorylation status in the presence of NRPa-47 at the half-maximal inhibitory anti-proliferative concentration previously reported on MDA-MB-231 (IC_50_ = 0.6 +/− 0.03 µM) [20]. Here, we showed that NRPa-47 significantly decreased tyrosine phosphorylation of both VEGF-R1 (20 to 40%) and VEGF-R2 (40 to 45%) from 5 min to 60 min on MDA-MB-231 (Figure 1A and Figure 1B, respectively). Of note, this inhibition is not due to intrinsic kinase activity inhibition as previously reported [20]. To strengthen this result, we followed the expression of both HIF-1α and VEGF-A_165_ mRNA as a negative feedback loop to validate the efficient abrogation of VEGF-R1 and VEGF-R2 phosphorylation mediated by NRPa-47. As expected, both HIF-1α and VEGF-A_165_ mRNA are reduced in the presence of its antagonist in a time-dependent manner with a maximum effect at 60 min (Figure 1C).

### 2.2. NRP-1/VEGF-Rs Downstream Signalling Modulation Induced by NRPa-47

To better understand the effect of NRPa-47 on MDA-MB-231, we extended our study using a biochemistry membrane platform targeting Mitogen-Activated Protein Kinase (MAPK) and downstream kinases (Figure 1D–E). Surprisingly, NRPa-47 did not negatively modulate MAPK such as extracellular signal-regulated kinases (ERK-1/-2) and c-Jun N-terminal kinases (JNK-1/-2/-3/-pan) pathways but contributed to their significant hyperphosphorylations at 10 min and at 60 min of drug exposure, respectively (Figure 1F,I). These MAPK hyperphosphorylations were in accordance with an increase in phosphorylation of their downstream substrates, such as p90 ribosomal S6 kinase (RSK-2), observed at 60 min (Figure 1G). However, RSK-1 remained unchanged (Figure 1G). Furthermore, phosphorylations of AKT-1/-2/-3/-pan were upregulated, as well as its downstream p70 ribosomal S6 kinase (p70S6K) (Figure 1F and Figure 1K, respectively).

In summary, even if the dephosphorylation of VEGF-R1/-R2 has been induced by NRPa-47, their downstream kinases became hyperphosphorylated. In front of these intriguing results, we focused our attention on the third MAPK, which consists of the p38 pathway, including p38α, p38β, p38γ, and p38δ. In this pathway, no significant variation in p38 phosphorylation has been observed except for p38α, which is significantly decreased at 60 min (Figure 1J). p38α downstream substrates, including small heat shock protein 27 (HSP27) and mitogen- and stress-activated protein kinase 2 (MSK2), were consequently dephosphorylated (Figure 1K). In addition, only the GSK-3β phosphorylation was affected (Figure 1H) due to the p38α phosphorylation defect but not to the ERK/AKT pathways. Taken together, NRPa-47 induces phosphorylation of ERK, JNK, and AKT pathways but inhibits p38α phosphorylation and its downstream kinases.

### 2.3. Modified-NRPa-47 (NRPa-48) and Its Own Effect on NRP-1/VEGF-Rs Downstream Signalling

**Figure 2:**
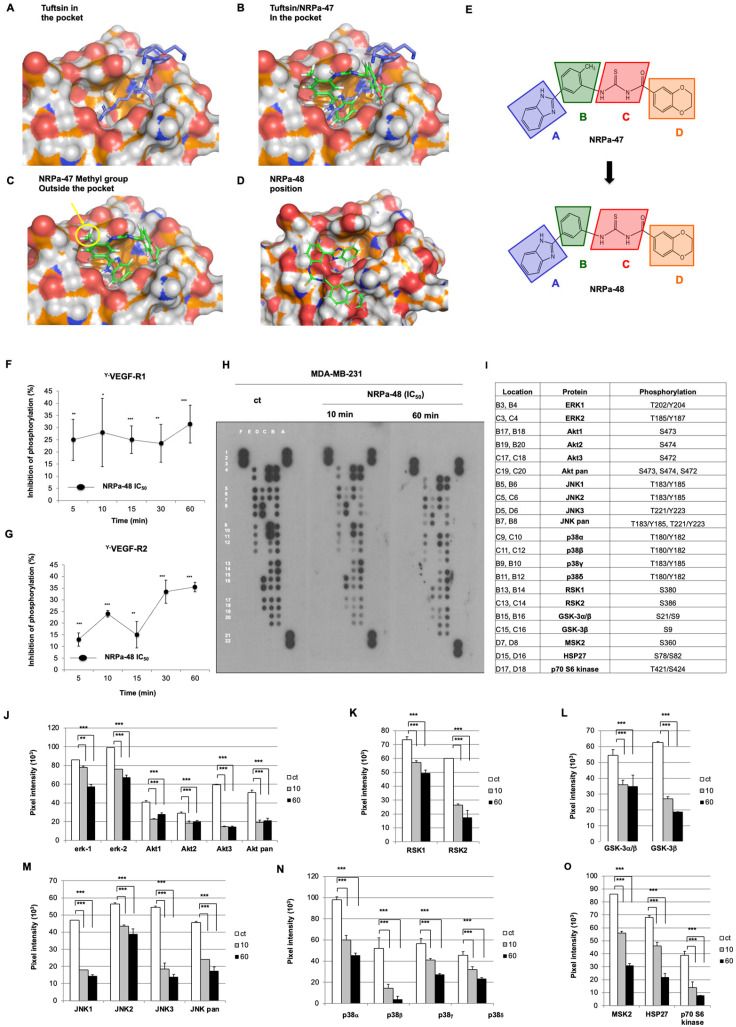
NRPa-48- and NRPa-47-derived mechanism of action on downstream VEGF signalling. (**A**–**D**) Tuftsin (**A**), Tuftsin/NRPa-47 (**B**), NRPa-47 (**C**), and NRPa-48 (**D**) predicting the docking model in the NRP-1 b1-domain. These dockings show the NRPa-47 methyl group localisation outside the pocket and highlight small divergences in the NRPa-47/NRP-1 b-1 domain and NRPa-48/NRP-1 b-1 domain interaction patterns probably induced by the “NRPa-47 magic methyl”. (**E**) NRPa-47 and NRPa-48 structural representations. Benzimidazole core (**A, blue**) is connected to a methylbenzene (**B, green**) linked to a benzodioxane motif (**D, orange**) through a carboxythiourea spacer (**C, red**). Methyl is suppressed from the methylbenzene (**B**) to generate the NRPa-48 compound. (**F**,**G**) Total tyrosine VEGF-R1 (Y-VEGF-R1) (**F**) and VEGF-R2 (Y-VEGF-R2) (**G**) phosphorylation inhibition induced by NRPa-48 (IC_50_ = 0.4 µM) on MDA-MB-231 in a time-dependent manner (5 to 60 min) using specific ELISA. Curves represent means +/− SD of at least three separate experiments, each performed in triplicate. (**H**) ECL developed the MAPK protein array film of untreated (control) and NRPa-48-treated MDA-MB-231 at short-time drug exposure (10 and 60 min). The following positions, A1/A2, A21/A22, and F1/F2, were positive controls; E3/E4, E5/E6, E7/E8, E9/10, and E11/E12 were antibody detection controls; and E13/14 were negative controls (background). Images are representative of at least two independent experiments for each condition. (**I**) Location map of phosphorylated proteins targeted in the MAPK protein array and their respective tyrosine (Y), serine (S), and threonine (T) phosphorylation positions detected. (**J**–**O**) Histograms show pixel intensity of untreated (ct, white histograms), NRPa-48-treated (IC_50_ = 0.4 µM) MDA-MB-231 at 10 (grey histograms) and 60 min (black histograms) of ERK-1/-2 and AKT-1/-2/-3/-pan (**J**), RSK-1/-2 (**K**), GSK-3α/β and GSK-3β (**L**), JNK-1/-2/-3/-pan (**M**), p38α/p38β/p38γ/p38δ (**N**), MSK2/HSP27/p70S6kinase (**O**) proteins. Histograms represent means ± SD of the previous representative selected experiment analysed using Image-J 1.52q software to quantify pixel intensity. (*, *p* < 0.05; **, *p* < 0.01; ***, *p* < 0.001).

Faced with these intriguing results between the dephosphorylation of VEGF-R and the hyperphosphorylation of downstream signalling mediated by NRPa-47, we performed a new structural docking analysis. To this end, we used the NRP-1 b1 defined-domain pocket by the tuftsin docking (Figure 2A). Using this docking, we superimposed tuftsin docking to NRPa-47 (Figure 2B); we observed that the methyl group of NRPa-47 is located outside the pocket (Figure 2C) and may constrain geometrically the unconventional carboxythiourea linker in an unexpected way. Thus, we decided to remove this methyl group in order to study this structurally related new compound called NRPa-48 (Figure 2E). The suppression of the methyl group changed the conformational position of NRPa-48 in the B1 domain (Figure 2D).

The relevance of the methyl group in drug candidates has been recently reviewed by Pinheiro et al. [23] and in the 2010s by Barreiro and coworkers [24]. It is definitely established that this functional group has a strong impact both on the pharmacophore-target interactions (it allows changes in the hydrophobic effects and in the Van der Waals interactions, and it may change the repartition of water molecules in the interacting pocket) and on the drug’s bioavailability (modification of the pharmacodynamic and pharmacokinetic effects), solubility and tissue permeability. It is also known that the methyl-induced steric hindrance may have an impact on the overall drug’s conformation and, in turn, on its global bioactivity. This has been observed during the development of the anti-cancer agent Imatinib [24,25]. To date, approximately 70% of the top-selling commercialised drugs bear such methyl (e.g., the anti-inflammatory ibuprofen; suvorexant, a drug used to treat insomnia; and imatinib, an anti-cancer agent) [26].

The ability of NRPa-48 to inhibit phosphorylation of both VEGF-R1 and VEGF-R2 has also been investigated at the half-maximal inhibitory anti-proliferative concentration previously reported on MDA-MB-231 (IC_50_ = 0.4 +/− 0.2 µM) [22]. As expected, NRPa-48 significantly exerted a tyrosine VEGF-R1 and VEGF-R2 kinase inhibitory capacity (Figure 2F and Figure 2G, respectively) but with a lower efficiency than NRPa-47 (Figure 1A,B). Despite this fact, NRPa-48 is also more efficient than NRPa-47 (0.6 vs. 0.4 µM) to block MDA-MB-231 proliferation [22]. Thus, these results prompted us to investigate its role in VEGF-Rs downstream signalling.

In contrast to NRPa-47, NRPa-48 significantly inhibited all tested MAPK (Figure 2H,I) such as ERK-1/-2, JNK-1/-2/-3/pan, and p38α/p38β/p38γ/p38δ, in a time-dependent manner (Figure 2J, Figure 2M, and Figure 2N, respectively). Their own respective downstream kinase substrates, such as RSK-1/RSK-2, MSK-2, HSP-27, and GSK-3α/β, were also inhibited in a time-dependent manner (Figure 2K,L,O). In addition, the AKT pathway, including AKT-1/-2/-3/-pan, was also inhibited from 10 min until 60 min (Figure 2J). Taken together, methyl group removal from the NRPa-47 chemical structure conferred to NRPa-48 an intriguing efficiency to block the activity of all MAPK and downstream kinases studied, induced by VEGF-A_165_.

**Figure 3:**
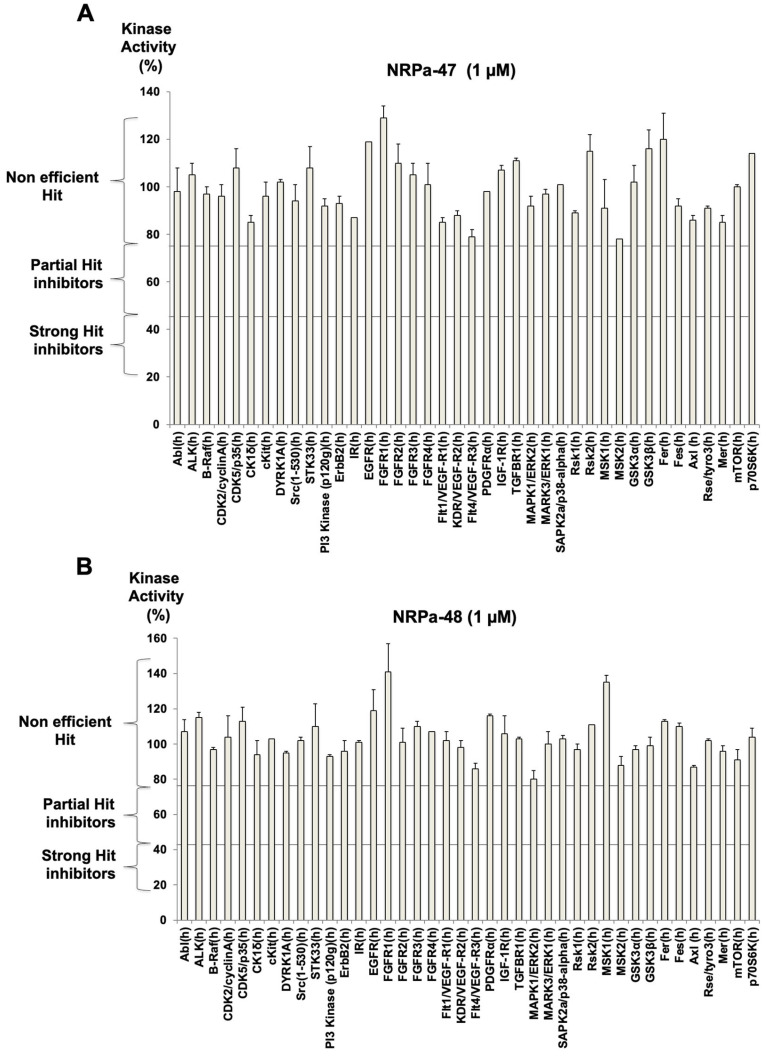
NRPa-47 and NRPa-48 protein kinase profiling. Protein kinase profiling of NRPa-47 (**A**) and NRPa-48 (**B**) was performed at 1 µM on a selection of 40 kinases, including Neuroplin-1 co-receptors and related biochemical kinase signalling observed during this study.

To strengthen the specificity of our hits, the activity of NRPa-47 and NRPa-48 was evaluated in vitro on 40 selected kinases among growth factor receptors, cell cycle kinases, insulin receptors, etc. None of these were significantly affected by both hits since kinase activity results remain confined in the non-efficient hits analysis section (Figure 3A,B).

Particularly, no kinase activity decreases in NRP co-receptors such as VEGF-R1/-R2/-R3, TGF-ß1-R1, FGF-R1/-R2/-R3/-R4, and EGF-Rs, or on VEGF-Rs downstream signalling has been observed (Figure 3A,B). More importantly, none of the in vitro tested kinase activity is blocked by these hits, in contrast to the observed kinase phosphorylation modulation induced by these hits in treated tumour cells. Thus, this test reinforced the impact of these hits on the inhibition of the cancer therapeutic key factor p38α, which is a specific downstream consequence of this treatment.

### 2.4. Apoptotic Pathway Induced by NRPa-47

**Figure 4:**
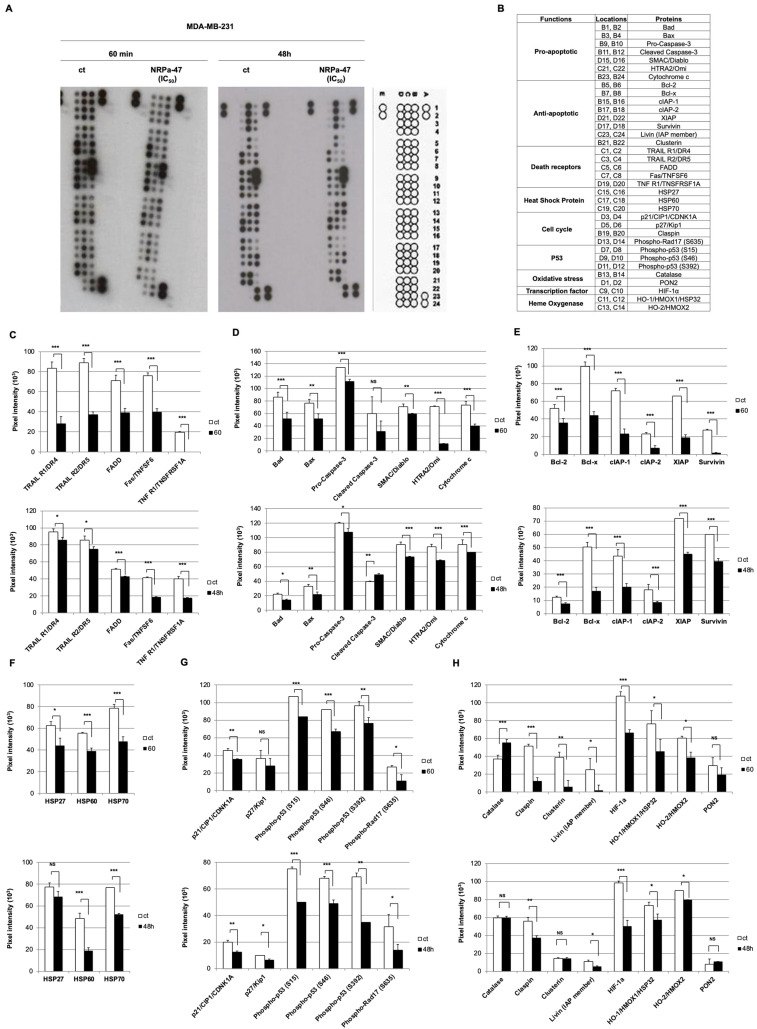
Apoptosis pathways regulation by NRPa-47. (**A**) ECL developed an apoptosis protein array film of untreated (control) and NRPa-47-treated MDA-MB-231 at short (10 min) and long (48 h) drug exposure with a position map. The following positions, A1/A2, A23/A24, and E1/F2, were positive controls, and D23/24 were negative controls (background). Images are representative of at least two independent experiments for each condition. (**B**) Location map of pro-apoptotic, anti-apoptotic, death receptor, heat shock protein, cell cycle, P53 pathway, oxidative stress, transcriptional factor, and heme oxygenase targeted in apoptosis protein array (letters and numbers indicate spot position). (**C-H-M**) Histograms show pixel intensity of untreated (ct, white histograms), NRPa-47-treated (IC_50_ = 0.6 µM) MDA-MB-231 at 60 min (grey histograms) and 48 h (black histograms) of pro-apoptotic (Bad, Bax, SMAC/Diablo, HTRA2/Omi, pro-caspase 3, caspase 3, and cytochrome c) (**C**), anti-apoptotic (Bcl-2, Bcl-x, cIAP-1, cIAP-2, XIAP, Survivin) (**D**), cell death receptors (TRAIL-R1/DR4, TRAIL-R2/DR5, FAS/TNFSF6, TNF-R1/TNSFRSF1A, FADD (**E**), heat shock proteins (HSP-27, HSP-60, HSP-70) (**F**), cell cycle and P53 pathway (**G**) and anti-apoptotic (Livin, Clusterin), oxidative stress (catalase, PON2), transcription factor (HIF-1α) and heme oxygenase (HO-1/HMOX1/HSP32, HO-2/HMOX2) (**H**) proteins. Histograms represent means ± SD of the previous representative selected experiment analysed using Image-J 1.52q software to quantify pixel intensity. (*, *p* < 0.05; **, *p* < 0.01; ***, *p* < 0.001; NS = not significant).

As both NRPa-47 and NRPa-48 exerted differential regulation of MAPK phosphorylation, we investigated the influence of this differential effect on cell death. Thus, we focused our study on NRPa-induced apoptosis cascades. To unravel this mechanism of action, we performed an apoptosis proteome array experiment at a short (60 min) and long (48 h) time (Figure 4A,B). We first analysed cell death receptors such as TRAIL-R1/DR4, TRAIL-R2/DR5, FAS/TNFSF6, TNF-R1/TNSFRSF1, and their adaptor protein FADD. All of these parameters were drastically down-modulated at 60 min and less at 48 h (Figure 4C). This result indicated that cell death induced by NRPa-47 seemed not to be dependent on cell death receptors. In addition, no pro-apoptotic proteins, including Bad, Bax, SMAC/Diablo, HTRA2/Omi, and cytochrome c, have been induced by NRPa-47; in contrast, a rapid decrease in these proteins was observed at short time drug exposure and remained decreased at the long time, except for the caspase-3 cleavage induction (Figure 4D). However, the anti-apoptotic proteins such as Bcl-2, Bcl-x, cIAP-1, cIAP-2, XIAP, Survivin, Livin, and Clusterin, as well as heat shock proteins (HSP-27, HSP-60, and HSP-70), were significantly reduced at 60 min and remained decreased at 48 h (Figure 4E,F,H).

Furthermore, NRPa-47 globally induced a reduction in cell cycle protein expression such as p21/CIP1/CDNK1A, p27/kip1, and phosphorylation of Rad-17 (Figure 4G). Moreover, all phosphorylation sites of the p53 protein were inhibited at 60 min (Figure 4G). Surprisingly, NRPa-47 induced rapid oxidative stress revealed by catalase induction but not the serum paraoxonase/arylesterase 2 (PON-2) at 60 min (Figure 4H).

In conclusion, NRPa-47 induced down-modulation of pro-apoptotic and anti-apoptotic proteins as well as cell death receptors. However, NRPa-47 rapidly led to an oxidative stress reflected by the catalase induction. In addition, expression of the inducible (HO-1/HMOX1/HSP32) and the constitutive (HO-2/HMOX2) heme oxygenase forms were both reduced at 60 min (Figure 4H). The cell death might be due to the decrease in both HIF-1α and Survivin (Figure 4H and Figure 4E, respectively).

### 2.5. Mechanism of NRPa-48-Induced Cell Death

**Figure 5:**
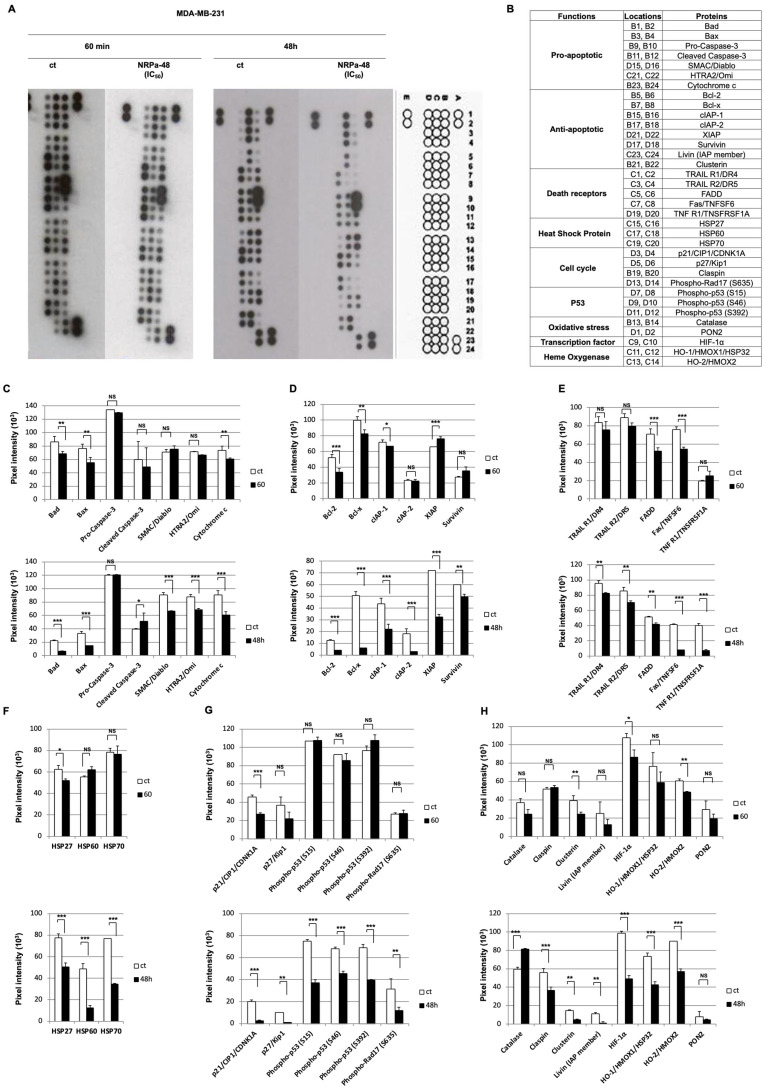
Apoptosis pathways regulation by NRPa-48 (IC_50_ = 0.4 +/− 0.2 µM). (**A**) ECL developed apoptosis protein array film of untreated (control) and NRPa-48-treated MDA-MB-231 at short (60 min) and long (48 h) time drug exposure with a position map (letters and numbers indicate spot position). The following positions, A1/A2, A23/A24, and E1/F2, were positive controls, and D23/24 were negative controls (background). Images are representative of at least two independent experiments for each condition. (**B**) Location map of pro-apoptotic, anti-apoptotic, death receptor, heat shock protein, cell cycle, P53 pathway, oxidative stress, transcriptional factor, and heme oxygenase targeted in apoptosis protein array. (**C-H-M**) Histograms show pixel intensity of untreated (ct, white histograms), NRPa-48-treated (IC_50_ = 0.4 +/− 0.2 µM) MDA-MB-231 at 60 min (grey histograms) and 48 h (black histograms) of pro-apoptotic (Bad, Bax, SMAC/Diablo, HTRA2/Omi, pro-caspase 3, caspase 3, and cytochrome c) (**C**), anti-apoptotic (Bcl-2, Bcl-x, cIAP-1, cIAP-2, XIAP, Survivin) (**D**), cell death receptors (TRAIL-R1/DR4, TRAIL-R2/DR5, FAS/TNFSF6, TNF-R1/TNSFRSF1A, FADD (**E**), heat shock proteins (HSP-27, HSP-60, HSP-70) (**F**), cell cycle, and P53 pathway (**G**) and anti-apoptotic (Livin, Clusterin), oxidative stress (catalase, PON2), transcription factor (HIF-1α), and heme oxygenase (HO-1/HMOX1/HSP32, HO-2/HMOX2) (**H**) proteins. Histograms represent means ± SD of the previous representative selected experiment analysed using Image-J 1.52q software to quantify pixel intensity. (*, *p* < 0.05; **, *p* < 0.01; ***, *p* < 0.001; NS = not significant).

To clarify the opposite effect of NRPa-48 compared to NRPa-47 on MAPK regulation, we extended our study to unravel its mechanism of action on the apoptotic pathway (Figure 5A,B). In contrast to the early NRPa-47 effect, NRPa-48 exerted a delayed effect on the apoptosis pathway regulation, as observed at 48 h, but not at 60 min (Figure 5C–H). Pro-apoptotic proteins such as Bad, Bax, SMAC/Diablo, HTRA2/Omi, and cytochrome c were significantly decreased, except for the caspase-3 cleavage induction (Figure 5C). The anti-apoptotic proteins such as Bcl-2, Bcl-x, cIAP-1, cIAP-2, XIAP, Survivin, Livin, Clusterin, the heat shock proteins (HSP-27, HSP-60, HSP-70), as well as the cell death receptors such as TRAIL-R1/DR4, TRAIL-R2/DR5, FAS/TNFSF6, TNF-R1/TNSFRSF1A, and their adaptor protein FADD were also down-modulated (Figure 5D–F,H). NRPa-48 induced a reduction in cell cycle protein expressions such as p21/CIP1/CDNK1A, p27/kip1, phosphorylation of Rad-17, and claspin (Figure 5G and Figure 5H, respectively). In addition, all phosphorylation sites of the p53 protein were inhibited (Figure 5G). In contrast to NRPa-47, NRPa-48 induced late oxidative stress, as demonstrated by the high level of catalase; however, PON-2 level remained unchanged (Figure 5H). Interestingly, NRPa-48 has the capacity to inhibit HIF-1α expression as previously observed for NRPa-47 (Figure 5H and Figure 4H, respectively). In addition, expression of the inducible (HO-1/HMOX1/HSP32) and the constitutive (HO-2/HMOX2) heme oxygenase forms were both reduced at 48 h (Figure 5H).

Taken together, both NRPas exerted similar down-modulation of proteins involved in apoptosis and induced oxidative stress. More interestingly, the most important proteins modulated in this pathway by NRPas were HO-1/HMOX1/HSP32, Survivin, and HIF-1α.

In conclusion, even if NRPa-47 and NRPa-48 did not have the same effect on MAPK signalling, both conducted the treated cell to the cell death programme in a similar manner but with different timing.

### 2.6. In Vivo Anti-Tumour Activity of NRPa-48 on Xenografted-NOG Mice

**Figure 6:**
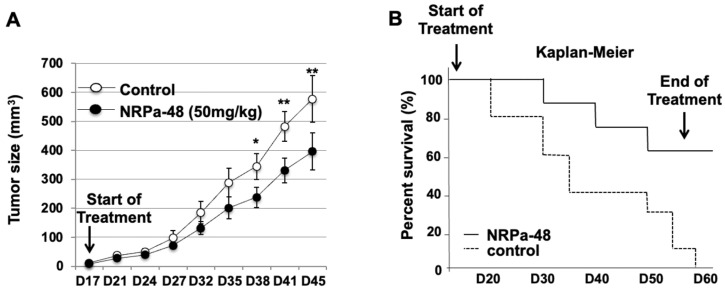
In vivo efficacy of NRPa-48. (**A**) NRPa-48 treatment (50 mg/kg) delayed tumour growth compared to control (PBS). Growth curves from MDA-MB-231 tumours in mice treated with NRPa-48 (n = 10) or vehicle (PBS) (n = 10). Tumour size measurement was interrupted when 50% of the animals were dead. (**B**) NRPa-48 (50 mg/kg) significantly enhances the survival of mice bearing MDA-MB-231 tumours. Kaplan–Meier survival curves (p) were determined using ANOVA (*p* = 0.008). Data are representative of 3 separate in vivo experiments. (*, *p* < 0.05; **, *p* < 0.01).

In vivo experiments were performed using NRPa-48 on MDA-MB-231-xenografted NOG mice to compare its efficiency on tumour growth inhibition to NRPa-47, as we already reported. [20] Mice were divided into two distinct groups; the first one was treated by force-feeding with NRPa-48 at 50 mg/kg three times a week, and the remaining group received the vehicle only (negative control). Interestingly, the treated animals did not show any loss of weight, suggesting that, at this concentration, NRPa-48 exhibits no acute toxicity. At Day 38 (21 days after starting treatment), tumour growth was strongly reduced by NRPa-48 (Figure 6A), since the tumour size was reduced by approximately 29% compared to the reference group (*** *p* < 0.001). The 50 mg/kg group remained largely efficient at Day 45 to exhibit a 34% tumour size reduction (Figure 6A). More interestingly, survival significantly increased when mice were treated with NRPa-48 at 50 mg/kg compared to the control group (Figure 6B). Thus, median survival was at 35 days for the animals in the control group and at over 56 days for the animals treated with NRPa-48 (*p* = 0.008) (Figure 6B). Taken together, in vivo tumour growth inhibition mediated by NRPa-48 is efficient, and 62% of treated mice at the end of treatment remained alive.

## 3. Discussion

The development of NRPas brought new tools for cancer treatment and the knowledge of biochemical pathways involved in this process. In this report, we observed that a very small structural change in the structure of two structurally related NRPas (NRPa-47 and NRPa-48), namely the suppression of a “magic-methyl” group, led to changes in the binding modes at the NRP-1 b1 domain. The overall antagonist conformational modification may be due to the methyl-related impairment of the core’s flexibility, as observed during the development of the anti-cancer agent imatinib [25]. These structural restrictions induced, in turn, major changes in the downstream signalling pathways, even if the average cytotoxicity of the two antagonists remains equivalent.

**Figure 7:**
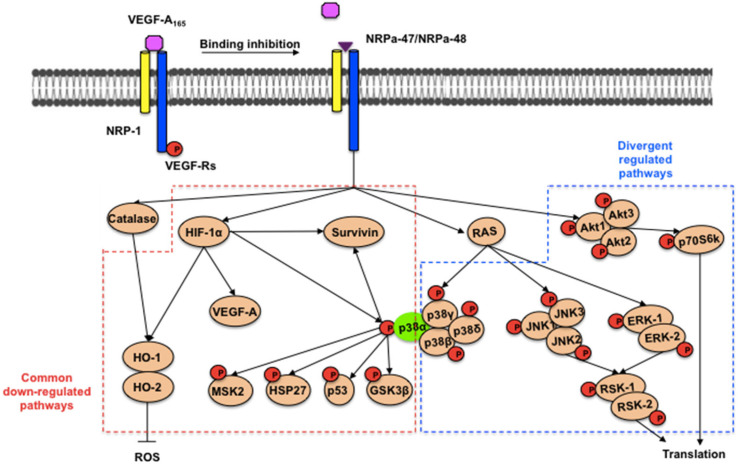
Schematic proposal of both NRPas mechanisms of action. VEGF-A_165_ binding to the functional NRP-1/VEGF-Rs complex was inhibited in the presence of neuropilin antagonists (NRPa-47 and NRPa-48). VEGF-Rs Tyr phosphorylation was inhibited by NRPa. Both NRPas exerted high oxidative-induced stress (catalase) and had a common down-regulated pathway (HIF-1α, heme oxygenase HO-1/HMOX1/HSP32 and HO-2/HMOX2 forms, phospho-heat shock protein 27 (HSP-27), VEGF-A_165_, Survivin, phospho-mitogen- and stress-activated protein kinase 2 (MSK2), phospho-p53, phospho-GSK-3β, and phospho-p38α). Both NRPas also mediated opposite regulating signalling (phospho-AKT, phospho-p70S6k, phospho-JNK-1/-2/-3, phospho-ERK-1/-2, phospho-p38β/γ/δ kinases, and RSK-1/-2.

NRP-1 inhibitors rapidly inhibit the HIF-1α protein and mRNA expression as well as the VEGF mRNA and thus may contribute to creating an interference with the autocrine HIF-1α/VEGF feedback loop. This result is very intriguing since HIF-1α is an important cancer drug target [27]. In this context, NRPas may induce tumour cell starvation for VEGF and compromise their growth and survival. In addition, high oxidative stress reflected by the increase in catalase expression is induced at 60 min for NRPa-47 and at 48h for NRPa-48. Even if both NRPas are capable of inducing dephosphorylation of VEGF-R’s tyrosine kinase, their downstream targets are not similarly affected. The main difference observed for the two NRPas is restraint to the MAPK (ERK, JNK, and p38 except p38α) regulation, which phosphorylations are increased by NRPa-47 and decreased by NRPa-48 (Figure 7). A similar difference is observed in the AKT pathway and its downstream target (p70S6kinase). Future investigations are needed to identify and/or clarify the alternative downstream pathway-mediated MAPK phosphorylation in this context.

Nevertheless, this opposite effect on MAPK regulation, leading to hyperphosphorylation as well as dephosphorylation, may provide the tumour cell death. No significant apoptotic pathways emerged between both, even if the down-modulation of death receptors, pro-apoptotic and anti-apoptotic proteins may cause an apoptotic/survival imbalance, which may also lead to cell death. The most pivotal events reliable to the apoptosis induction are the decrease in Survivin expression, the induction of oxidative stress (catalase), the down-regulation of heme oxygenase and the down-modulation of HIF-1α. Other HIF-1α inducers, such as HO-1/HMOX1/HSP32, inhibited expression as the p38α phosphorylation, which is also an activator of HO-1. The p38α pathway inhibition occurs with the p53 dephosphorylation, the defect of HSP27, GSK-3ß and MSK2 phosphorylation, as well as the down-modulation of Survivin. Disruption of Survivin expression leads to increased apoptosis and decreased tumour growth.

Taken together, this report highlighted the pivotal role of p38α in the cell signalling cascade mediated by NRPa. Several studies report p38α as a drug target to develop specific inhibitors to treat cancer relying on p38 MAPK activity for progression [28,29]. The association of p38α inhibitors with DNA-damaging chemotherapy may trigger cancer cell death by the impairment of p38α-mediated cell cycle arrest and DNA repair mechanisms [30].

**Figure 8:**
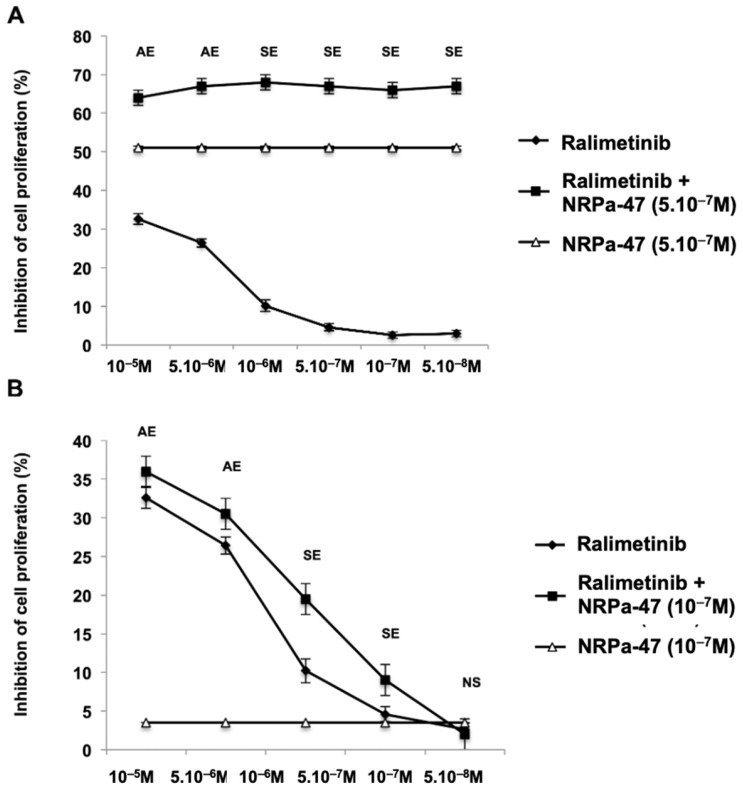
NRPa/Ralimetinib^®^ association increased anti-breast cancer cell proliferation. A concentration range of Ralimetinib^®^ is tested alone or in association with NRPa IC_50_ (**A**) or sub-optimal NRPa IC_50_ (0.1 µM) (**B**) on MDA-MB-231 cell proliferation. The NRPa IC_50_/Ralimetinib^®^ association showed an additive effect (AE) at high Ralimetinib^®^ concentration and a synergistic effect (SE) at low Ralimetinib^®^ concentration (**A**). Sub-optimal NRPa IC_50_/Ralimetinib^®^ association showed an additive effect (AE) at high Ralimetinib^®^ concentration and a synergistic effect (SE) at low Ralimetinib^®^ concentration. Data represent means ± SD of 3 separate experiments, each. (NS: Not significant).

More importantly, the association of NRPas with Ralimetinib^®^ (Phospho-p38α inhibitor) strengthened this hypothesis since the additional and/or synergistic effect of these drugs (depending on the dose used) significantly reduced breast cancer cell proliferation (Figure 8A,B). In summary, NRPas might be used alone or in association with a drug to treat cancer. This observation brought the newest interest for the development of NRPa.

## 4. Materials and Methods

### 4.1. Chemical Synthesis of Compound

Chemical reagents and solvents were purchased from Sigma Aldrich (Taufkirchen, Germany), Fluka (Charbonnieres, France) and Carlo Erba (Val-de-Reuil, France). NRPa-47 and NRPa-48 have been synthesised and characterised as previously reported by us [20,22].

### 4.2. Total RNA Preparation and RT-PCR

MDA-MB-231 RNA was extracted with the NucleoSpinRNA II kit (Macherey-Nagel, Hoerdt, France) and quantified using Nanodrop (ND-1000 spectrophotometer, Thermo Fisher Scientific, Waltham, MA, USA). 1 μg of each RNA sample was reverse-transcribed into cDNA using the iScript cDNA Synthesis Kit (Bio-Rad, Marnes-la-Coquette, France) following the manufacturer’s instructions. Specific primers were used as follows: VEGF-A_165_, 5′-GCCTTGCCTTGCTGCTCTAC-3′, and 5′-GCTGGCCTTGGTGAGGTTTG-3′; HIF-1α, 5′-CATTACCCACCGCTGAAACG-3′, and 5′-TTCACTGGGACTATTAGGCTC-3′; and RPLPO, 5′-CAT-TGC-CCC-ATG-TGA-AGT-C-3′, and 5′-GCT-CCC-ACT-TTG-TCT-CCA-GT-3′. PCR amplification was performed in a reaction mixture (25 μL) containing 200 μM of each dNTP, 1 μg of cDNA, 1 μM of primers, and 0.625 U of GoTaq DNA Polymerase (Promega, Charbonnieres, France) with 45 s of denaturation at 95 °C, 45 s of annealing at 60 °C and 1 min of extension at 72 °C for 30 cycles. PCR products were separated by 1% agarose gel electrophoresis, stained with ethidium bromide (Sigma, Taufkirchen, Germany) and analysed using the Gel Doc 2000 System (Bio-Rad, Marnes-la-Coquette, France).

### 4.3. Proteome Profiler Arrays

MDA-MB-231 cells were incubated in the presence or absence of NRPa-47 or NRPa-48 compounds (IC_50_), and protein lysates were prepared and quantified as previously described. [31,32] Biochemical signalling detection was evaluated by using a human proteome profiler array (human phosphokinase array and human apoptosis array) according to the manufacturer’s instructions (R&D systems, Lille, France). Briefly, capture and control antibodies were spotted in duplicate on nitrocellulose membranes. Cellular extracts were incubated overnight on the membrane, washed to remove unbound proteins, and followed by incubation with a cocktail of biotinylated detection antibodies. Streptavidin-HRP and chemiluminescent detection reagents were applied, and the signal intensity corresponding to the amount of protein bound was measured at each capture spot using ImageJ 1.52q software.

### 4.4. Cell Culture Conditions

The human aggressive and metastatic estrogenR-/progesteroneR-/Her2- triple negative breast cancer cell line (MDA-MB-231) purchased from the ATCC (Molsheim, France) was plated in 200 µL/well in 96-well plates at 10.10^3^ cells/well and was treated or not with NRPa-47 (IC_50_), NRPa-48 (IC_50_), and Ralimetinib^®^ alone or in combination at different concentrations. WST-1 (Roche^®^, Meylan, France) was added for 1–2 h, then optical density was analysed with a microplate reader (Microplate Manager 5.2, Bio-Rad, Marnes-la-Coquette, France) at 490 nm to determine the cell viability. For each compound, the IC_50_ value was determined from a sigmoid dose–response curve using GraphPad Prism version 7 (GraphPad Software, San Diego, CA, USA).

### 4.5. VEGF-R Kinase Assay

The cells were cultured in the presence of NRPa-47 or NRPa-48 at their IC_50_ for 5 to 60 min, and then the MDA-MB-231 lysates were used to detect total VEGF-R1 and VEGF-R2 tyrosine phosphorylation using the ELISA assay (R&D system).

### 4.6. Molecular Docking

The binding site has been defined at 4 Å around the co-crystallised tuftsin bound to NRP-1 (PDB code 2ORZ) [33]. Consensus molecular docking was performed using Surflex dock v2.5. [34] and ICM-VLS-v3.4. [35] Surflex dock is based on a modified Hammerhead fragmentation/reconstruction algorithm to dock compounds flexibly into the binding site. The query molecule is decomposed into rigid fragments that are superimposed on the Surflex Protocol, i.e., molecular fragments covering the entire binding site. The docking poses were evaluated by an empirical scoring function. ICM is based on Monte Carlo simulations in internal coordinates to optimise the position of molecules using a stochastic global optimisation procedure combined with pseudo-Brownian positional/torsional steps and fast local gradient minimisation. The docking poses were evaluated using the ICM VLS empirical scoring function.

### 4.7. Protein Kinase Profiling

NRPa-47 and NRPa-48 specificity profiling assays were carried out at Eurofins Pharma Discovery Services (Dundee, UK) for protein kinase profiling against a selected panel of 40 protein kinases. Results of protein kinases assayed at 1 µM of each NRPas are presented as a percentage of kinase activity in DMSO control reactions.

### 4.8. In Vivo Xenografted-Tumour Mouse Model

The protocol was approved by the INSERM Institutional Care and Use Committee according to the European Communities Council Directive using TrGET, a preclinical assay platform, CRCM, INSERM U1068-Institut Paoli-Calmette, Marseille, France. NOD/scid/IL-2Rγ-/- (NOG) female mice were bred and housed in pathogen-free conditions in accordance with the Federation of European Animal Associations (FELSA) guidelines. MDA-MB-231 cells were washed twice in PBS and resuspended in DMEM. Subsequently, cells were injected subcutaneously into NOG mice (6–7 weeks old) at the concentration of 2.10^6^ cells/200 µL. Mice were randomly divided into different groups (10 mice/group). Mice then received NRPa-48 (50 mg/kg) or vehicle using force-feeding every three days for 39 days. Tumour growth and body weight were measured every three days during the treatment. Mice were weighed regularly to assess the toxicity of the treatment, and the tumours were measured with callipers (width × width × length × Pi/6) to determine growth.

### 4.9. Statistical Analysis

Data are expressed as the arithmetic mean +/− SD of at least three different experiments. The statistical significance of results was evaluated by ANOVA, with probability values * *p* < 0.05, ** *p* < 0.01, and *** *p* < 0.001 being considered as significant.

## 5. Conclusions

The development of combination therapies, combining NRP-1 inhibitors with other anti-cancer treatments, could improve the effectiveness of existing treatments. Drug combinations could overcome resistance to current treatments and improve patient outcomes.

Nevertheless, no data indicate the importance of the oncogenic status of tumour cells, such as the oncogenic RAS protein in the cell signalling mediated by NRP-1. This point should be investigated in the future to identify potential resistance to the NRPa treatment.

## Data Availability

No new data were created or analysed in this study. Data sharing is not applicable to this article.
